# Case Report: Bacteriophage-antibiotic therapy for extensively drug-resistant *Acinetobacter baumannii* in critically ill patient with respiratory infection

**DOI:** 10.3389/fmed.2025.1716306

**Published:** 2025-12-17

**Authors:** Jie Lin, Guiqin Dai, Lei Zhang, Pengli Xu, Pengfei Zhao, Yang Zhou, Hongzhou Lu, Mingbin Zheng

**Affiliations:** 1 National Clinical Research Center for Infectious Disease, Shenzhen Third People’s Hospital, Southern University of Science and Technology, Shenzhen, China; 2 Molecular Biology Research Center and Center for Medical Genetics, School of Life Sciences, Central South University, Changsha, China; 3 The Affiliated Dongguan Songshan Lake Central Hospital, Guangdong Medical University, Dongguan, China

**Keywords:** bacteriophage therapy, drug resistance, *Acinetobacter baumannii*, respiratory infection, pulmonary disease

## Abstract

Extensively drug-resistant *Acinetobacter baumannii* (XDR *A. baumannii*) poses a crucial challenge due to high mortality and limited therapies. Here, we report the successful application of phage-antibiotic synergy in a critically ill patient with two months of ineffective antibiotic treatment. The patient was diagnosed with severe pneumonia due to recurrent infection of XDR *A. baumannii*, causing severe pulmonary dysfunction. A nebulized phage inhalation combined with intravenous administration of polymyxin B, amikacin, and fosfomycin successfully brought about measurable clinical improvements in 8 days. The clearance of XDR *A. baumannii* in sputum cultures, coupled with decreased partial pressure of carbon dioxide, substantial absorption of bilateral pulmonary lesions, reduced density of residual infiltrates, and decreased pleural effusion in the patient, collectively confirmed therapeutic efficacy. Our case indicate that bacteriophage-antibiotic therapy is promising to prevent the emergence of resistant mutants and enhance antibacterial efficacy in patient with similar infections.

## 1 Introduction

*Acinetobacter baumannii* (*A. baumannii*) is a pathogenic bacterium with intrinsic phenotypic resistance to multiple antibiotics, and can easily acquire resistance genes (1). Drug-resistant *A. baumannii* infections are a vital cause of mortality and morbidity among immunocompromised patients with hospital-acquired and ventilator-associated pneumonia (2). These infections are highly prone to progression to lobar consolidation, bacteremia, and even septic shock, thereby leading elevated mortality rates (3, 4). Current treatment strategies for drug-resistant *A. baumannii* pneumonia primarily involve antibiotic combination medication and regimen optimization, which are based on drug susceptibility results (5, 6). Endorsed by the 2024 Infectious Diseases of America guidelines, sulbactam-durlobactam combined with background carbapenem therapy is the preferred regimen for carbapenem-resistant *A. baumannii* pulmonary infection (6). However, rapidly emerged bacterial resistance limit the antibiotic option, causing recurrent infections, prolonged clinical course, and severe lung function impairment (3). Thus, targeted and rapid therapies for drug-resistant bacterial infection are urgently needed.

Bacteriophages are viruses which precisely infect and lyse host bacteria through receptor-binding and enzymatic degradation mechanisms. With emerging drug resistance and their mechanism distinct from antibiotic, phages have gained renewed interest as an alternative or complementary therapeutic tool, particularly for treating drug-resistant bacterial infections (7, 8). Nevertheless, the lytic efficacy of phages is limited by excessive specificity and narrow antimicrobial spectrum, preventing comprehensive bacterial clearance. Due to reduced potential for resistance and broad spectrum activity, bacteriophage and antibiotic synergy have demonstrated great bactericidal potential against various drug-resistant clinical strains, including *Acinetobacter baumannii*, *Pseudomonas aeruginosa* and *Staphylococcus aureus* (9–12). Here, we report the combined use of phage and antibiotic for an elder in intensive care unit with severe *A. baumannii* pneumonia, who experienced recurrent infection after antibiotic therapy. The infection precipitated acute respiratory distress syndrome (ARDS) and subsequent pulmonary failure, which was severely compounded by pre-existing chronic obstructive pulmonary disease (COPD). The bacteriophage-antibiotic treatment yielded positive outcomes in 8 days, including resolution of pulmonary infiltration, reduced respiratory distress, and decreased levels of inflammatory factors. The clearance of extensively drug-resistant (XDR) *A. baumannii* in sputum revealed the effectiveness of the phage-antibiotic combinational therapy in eliminating drug-resistant bacterial infection and recovering crucial lung function.

## 2 Case presentation

**Figure 1:**
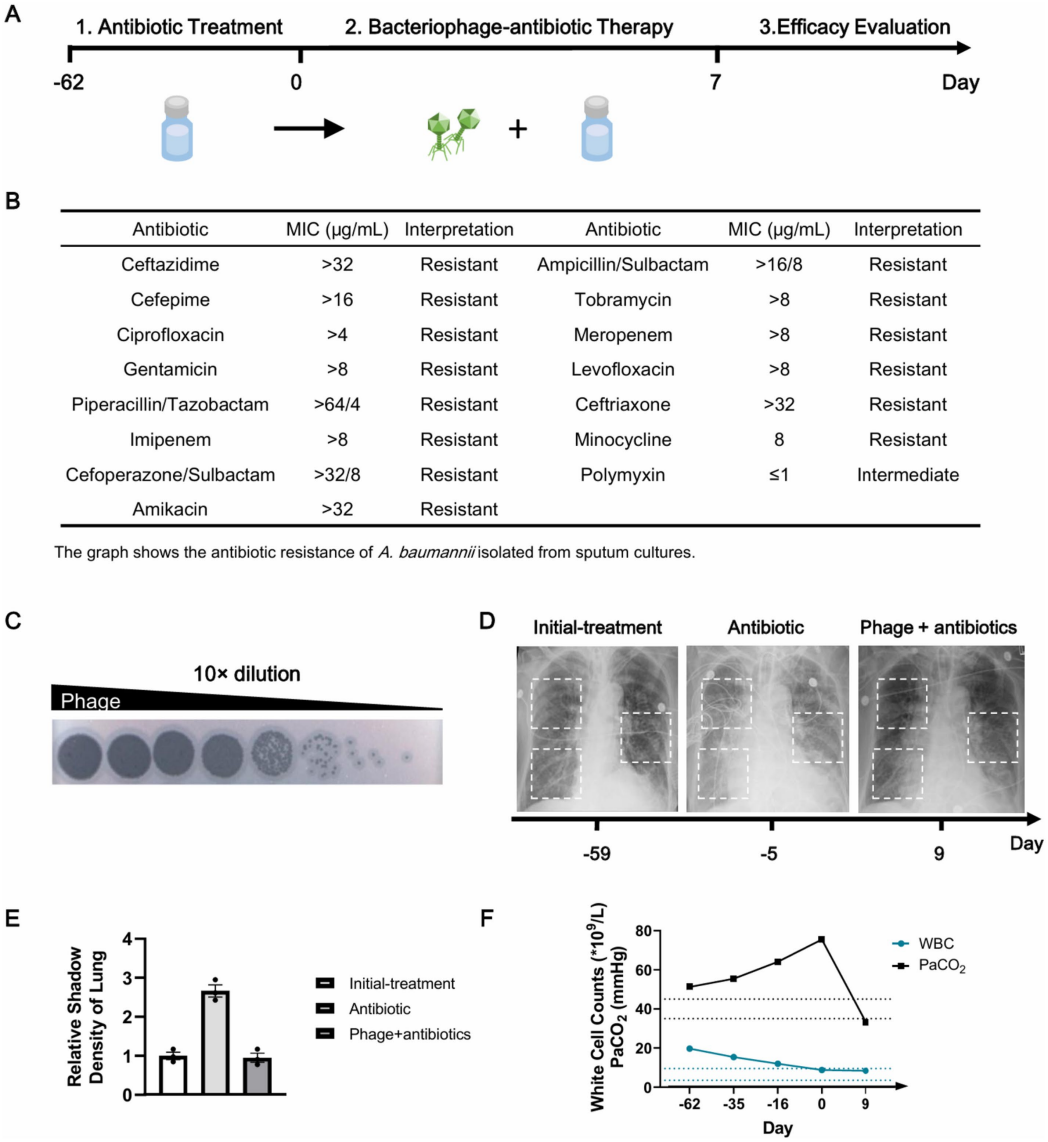
Bacteriophage-antibiotic therapy for extensively drug-resistant *A. baumannii*. **(A)** Timeline of the treatment. **(B)** *In vitro* antimicrobial susceptibility profile of *A. baumannii* isolate. **(C)** Susceptibility of *A. baumannii* isolates to phage determined by plaque assays. The titer of the bacteriophage was 1.25 × 10^10^PFU/mL. **(D)** The radiograph after continuous antibiotic treatment revealed progression of right-sided pleural effusion. Following 8 days of bacteriophage-antibiotic therapy, radiograph showed significant absorption of bilateral pulmonary lesions, reduced density of residual infiltrates and decreased pleural effusion. **(E)** The relative shadow density of chest X-ray at the white dotted regions. **(F)** The white cells count and PaCO_2_ of the patient. The reference range of the white cells count (WS/T 246—2005) and PaCO_2_ was shown in dashed lines.

The patient was a woman in her 80s with a history of hypertension, COPD, and bronchial asthma. She was admitted with a 20-day history of cough, phlegm, chest tightness, wheezing, dyspnea, and fever. Following the onset of infection-precipitated ARDS and subsequent pulmonary failure, she was mechanically ventilated under sedation and analgesia, with a Richmond Agitation-sedation Scale Score of −3 (deep sedation). The patient presented with an elevated partial pressure of carbon dioxide (PaCO_2_) of 51.2 mmHg, indicative of hypoventilation. Laboratory findings included a markedly elevated white blood cell count of 19.70 × 10⁹/L (neutrophils 92.1%, lymphocytes 4.2%, monocytes 3%), a normal platelet count of 329 × 10⁹/L, and a high C-reactive protein level of 203.4 mg/L. These results showed a severe inflammation and probable immune damage of the patient. Both the sputum culture from the day of admission and a prior culture from an external hospital identified *A. baumannii*. Initial chest X-ray revealed bilateral multifocal pulmonary infiltrates, with small left-sided pleural effusion, suggestive of severe bacterial pneumonia at admission. The patient underwent empirical antibiotic treatment with cefuroxime, polymyxin E, and linezolid, and subsequent antimicrobial susceptibility-guided therapy for 2 months (Figure 1A). The regimens are as follows: cefuroxime at 1.5 g every 8 h, polymyxin E at 150 mg every 12 h, and linezolid at 0.6 g every 12 h. The drug susceptibility result revealed an XDR phenotype of *A. baumannii* isolate, which represented resistance to multiple antibiotics such as ceftazidime, cefepime and ciprofloxacin (Figure 1B). The patient presented with refractory chronic infection and became exacerbated. Recurrently positive cultures for XDR *A. baumannii* and aggravated right-sided pleural effusion also indicated failure of the antibiotic therapy.

To address the recurrent infection of XDR *A. baumannii*, we initiated a phage cocktail aerosol inhalation combined with intravenous administration of antibiotics to enhance therapeutic efficacy. The bacteriophages were isolated from sewage water samples collected from Shenzhen Third People’s Hospital (self-screened). *In vitro* assays confirmed the strong lytic efficacy of the selected bacteriophage against clinical *A. baumannii* isolates obtained from the patient (Figure 1C). Genome sequencing analyses revealed that this lytic phage did not contain any virulence genes and has similarities with Acinetobacter phage Arbor (NCBI accession number, ON237674.1, 99.82% identity, 91% coverage). Nebulized phage was administered in 20-min sessions, twice a day (8 × 10^9^ PFU in 5 mL saline) *via* high-frequency nebulizer to eradicate the pathogen, with treatments operated in the morning and afternoon. Ethical approval was obtained from the Ethics Commission of Shenzhen Third People’s Hospital (Approval number, 2021–068-03). Simultaneously, intravenous antibiotics were administered according to the following regimen: polymyxin B at a loading dose of 1 million units, followed by 500,000 units every 12 h, amikacin at 200,000 units every 12 h, and fosfomycin at 8 g every 8 h. The combined therapy was continued for 8 days in the intensive care unit without any changes.

Following bacteriophage-antibiotic therapy, the mental state of patient significantly improved, with the Richmond Agitation-sedation Scale Score changing from −3 (deep sedation) on admission to −1 (drowsy but arousable) after treatment. Follow-up tests revealed significant declines in inflammatory markers, with the white blood cell count declined from 19.70 × 10⁹ to 8.39 × 10⁹ cells/L, and C-reactive protein levels dropped from 203.40 to 67.33 mg/L, which indicated progressive resolution of the infection (Figure 1F). Chest X-ray after bacteriophage-antibiotic treatment showed substantial absorption of bilateral pulmonary lesions, reduced density of residual infiltrates, and decreased pleural effusion (Figures 1D,E). This was further supported by improved alveolar ventilation, with PaCO₂ levels decreasing from 51.2 to 37.4 mmHg. Moreover, sputum cultures from 5 consecutive sets showed no pathogenic bacterial growth. The bacteriophage-antibiotic therapy achieved the rapid and effective rescue of the patient’s pulmonary function and inflammatory status, demonstrating a favorable therapeutic response.

## 3 Discussion

In this case, the elderly patient with a history of COPD developed ARDS and subsequent pulmonary dysfunction due to XDR *A. baumannii* infection, facing profound therapeutic challenges. Moreover, delayed initiation of targeted therapy at advanced disease stages limited the possibility for maximal benefit. Under these circumstances, pulmonary infection with XDR *A. baumannii* was particularly difficult to manage. Prolonged broad-spectrum antibiotic therapy yielded a suboptimal response, with neither clinical improvement nor microbiological clearance. Serial sputum cultures over an extended course of empiric antibiotic therapy confirmed recurrent infection of XDR *A. baumannii*. Even subsequent susceptibility-guided treatment failed, with recrudescence of XDR *A. baumannii* and evidence of progressive pulmonary disease, indicating incomplete bacterial clearance and reflecting the limitations of current antibiotic regimens.

Phages and antibiotics act *via* complementary mechanisms, with phages lysing bacteria and antibiotics suppressing planktonic and residual bacterial populations (13). The core advantages of combining phages with antibiotics are their synergistic antibacterial efficacy, reduced resistance potential and expanded bactericidal spectrum (11, 14). Compared with antibiotics, bacteriophages offer unique advantages, including targeted bactericidal activity, reduced off-target toxicity, and a lower propensity for resistance development (13, 15). Nevertheless, its lytic activity is limited by excessive specificity, and is often insufficient for complete bacterial eradication. The introduction of adjunctive phage therapy marked a turning point. In cases of phage treatment of *A. baumannii*, the combined use of phage and antibiotic may prevent the development of phage resistance and the mutation of drug susceptibility (16). Previous *in vitro* studies have demonstrated that combining phages with antibiotics can prevent the emergence of resistant mutants and enhance bactericidal efficacy (11, 17). In this case, the combined therapeutic strategy of phage and antibiotics (polymyxin B, amikacin, and fosfomycin) achieved synergistic bactericidal effect *in vitro*. The progressive clearance of XDR *A. baumannii* burden, paralleled by marked clinical improvement in radiographic findings, respiratory function, and systemic inflammation, all highlighted the rapid and effective antibacterial synergy of phage-antibiotic combination therapy. The rapid control of infection suggests the early deployment of such combination therapy. It may restrict resistance evolution and liberate patients with respiratory failure due to drug-resistant bacterial infection within a narrow therapeutic window.

Although bacteriophage-antibiotic therapy yielded favorable therapeutic responses in this patient, it still faces several challenges, including long-term stability, toxin release by lysed bacteria and optimal dosing regimens. Besides, the sequence and types of antimicrobial application are critical determinants of treatment success (11). For instance, study on methicillin-resistant *Staphylococcus aureus* (MRSA) infection indicated optimal effectiveness when phage treatment preceded antibiotics (18). While in our case, concurrent administration of phages and antibiotics achieved favorable outcomes. These differences underscore the complexity of phage-antibiotic interactions, which may range from synergistic to antagonistic effects depending on the bacterial strain and drug class (9–11, 19). Both therapeutic efficacy and potential adverse effects of phage-antibiotic interactions and dosing strategies require further mechanistic investigation.

## 4 Conclusion

Drug-resistant infections constrain antibiotic options, and rapid infection progression in patients with medical conditions presents major clinical challenges. Bacteriophages effectively lyse antibiotic-resistant bacteria, bypassing conventional antimicrobial resistance mechanisms. Timely synergistic therapy is crucial. The short-term treatment with phage and synergistic antibiotics successfully eliminated XDR *A. baumannii*, promptly halted disease progression.

## Data Availability

The original contributions presented in the study are included in the article/supplementary material, further inquiries can be directed to the corresponding authors.

## References

[1] Gao F Zhou R He Y Zhang Y Bao C Feng G. Bio-mimicking nanoparticle system facilitates sonodynamic-mediated clearance of extensively drug-resistant bacteria. ACS Biomater Sci Eng. (2025) 11:2988–3002. doi: 10.1021/acsbiomaterials.4c02455, 40294106

[2] Mohd Sazlly Lim S Zainal Abidin A Liew SM Roberts JA Sime FB. The global prevalence of multidrug-resistance among *Acinetobacter baumannii* causing hospital-acquired and ventilator-associated pneumonia and its associated mortality: A systematic review and meta-analysis. J Infect. (2019) 79:593–600. doi: 10.1016/j.jinf.2019.09.012, 31580871

[3] Rodríguez-Aguirregabiria M Lázaro-Perona F Cacho-Calvo JB Arellano-Serrano MS Ramos-Ramos JC Rubio-Mora E . Challenges facing two outbreaks of carbapenem-resistant *Acinetobacter baumannii*: from cefiderocol susceptibility testing to the emergence of cefiderocol-resistant mutants. Antibiotics. (2024) 13:784. doi: 10.3390/antibiotics13080784, 39200084 PMC11350900

[4] Lee Y-T Kuo S-C Yang S-P Lin Y-T Chiang D-H Tseng F-C . Bacteremic nosocomial pneumonia caused by Acinetobacter baumannii and *Acinetobacter nosocomialis*: a single or two distinct clinical entities? Clin Microbiol Infect. (2013) 19:640–5. doi: 10.1111/j.1469-0691.2012.03988.x, 22967204

[5] Bavaro DF Belati A Diella L Stufano M Romanelli F Scalone L . Cefiderocol-based combination therapy for “difficult-to-treat” gram-negative severe infections: real-life case series and future perspectives. Antibiotics. (2021) 10:652. doi: 10.3390/antibiotics10060652, 34072342 PMC8227820

[6] Cj K C G A-C U. *Acinetobacter baumannii* treatment strategies: a review of therapeutic challenges and considerations. Antimicrob Agents Chemother. (2025) 69:e0106324. doi: 10.1128/aac.01063-24 40631987 PMC12326985

[7] Köhler T Luscher A Falconnet L Resch G McBride R Mai Q-A . Personalized aerosolised bacteriophage treatment of a chronic lung infection due to multidrug-resistant *Pseudomonas aeruginosa*. Nat Commun. (2023) 14:3629. doi: 10.1038/s41467-023-39370-z, 37369702 PMC10300124

[8] Li N Li L He B Li D Jin W Wu Y . Personalized bacteriophage therapy for chronic biliary tract *Pseudomonas aeruginosa* infections. hLife. (2025) 3:275–83. doi: 10.1016/j.hlife.2025.03.004

[9] Yj C S K M S J K. Synergistic antimicrobial effects of phage vB_AbaSi_W9 and antibiotics against *Acinetobacter baumannii* infection. Antibiotics. (2024) 13:680. doi: 10.3390/antibiotics13070680, 39061362 PMC11273692

[10] Otava UE Tervo L Havela R Vuotari L Ylänne M Asplund A . Phage-antibiotic combination therapy against recurrent Pseudomonas septicaemia in a patient with an arterial stent. Antibiotics. (2024) 13:916. doi: 10.3390/antibiotics13100916, 39452183 PMC11504013

[11] Akturk E Pinto G Ostyn L Crabbé A Melo LDR Azeredo J . Combination of phages and antibiotics with enhanced killing efficacy against dual-species bacterial communities in a three-dimensional lung epithelial model. Biofilms. (2025) 9:100245. doi: 10.1016/j.bioflm.2024.100245, 40585316 PMC12206332

[12] Kunz Coyne AJ Bleick C Stamper K Kebriaei R Bayer AS Lehman SM . Phage-antibiotic synergy against daptomycin-nonsusceptible MRSA in an ex vivo simulated endocardial pharmacokinetic/pharmacodynamic model. Antimicrob Agents Chemother. (2024) 68:e0138823. doi: 10.1128/aac.01388-23, 38376187 PMC10989002

[13] Su J Tan Y Liu S Zou H Huang X Chen S . Characterization of a novel lytic phage vB_AbaM_AB4P2 encoding depolymerase and its application in eliminating biofilms formed by *Acinetobacter baumannii*. BMC Microbiol. (2025) 25:123. doi: 10.1186/s12866-025-03854-3, 40057696 PMC11889872

[14] Le S Wei L Wang J Tian F Yang Q Zhao J . Bacteriophage protein Dap1 regulates evasion of antiphage immunity and *Pseudomonas aeruginosa* virulence impacting phage therapy in mice. Nat Microbiol. (2024) 9:1828–41. doi: 10.1038/s41564-024-01719-5, 38886583

[15] Ling H Lou X Luo Q He Z Sun M Sun J. Recent advances in bacteriophage-based therapeutics: insight into the post-antibiotic era. Acta Pharm Sin B. (2022) 12:4348–64. doi: 10.1016/j.apsb.2022.05.007, 36561998 PMC9764073

[16] Aslam S Lampley E Wooten D Karris M Benson C Strathdee S . Lessons learned from the first 10 consecutive cases of intravenous bacteriophage therapy to treat multidrug-resistant bacterial infections at a single center in the United States. Open Forum Infect Dis. (2020) 7:ofaa389. doi: 10.1093/ofid/ofaa389, 33005701 PMC7519779

[17] Hong Q Chang RYK Assafiri O Morales S Chan H-K. Optimizing in vitro phage-ciprofloxacin combination formulation for respiratory therapy of multi-drug resistant *Pseudomonas aeruginosa* infections. Int J Pharm. (2024) 652:123853. doi: 10.1016/j.ijpharm.2024.123853, 38280500

[18] Loganathan A Bozdogan B Manohar P Nachimuthu R. Phage-antibiotic combinations in various treatment modalities to manage MRSA infections. Front Pharmacol. (2024) 15:1356179. doi: 10.3389/fphar.2024.1356179, 38659581 PMC11041375

[19] Łusiak-Szelachowska M Międzybrodzki R Drulis-Kawa Z Cater K Knežević P Winogradow C . Bacteriophages and antibiotic interactions in clinical practice: what we have learned so far. J Biomed Sci. (2022) 29:23. doi: 10.1186/s12929-022-00806-1, 35354477 PMC8969238

